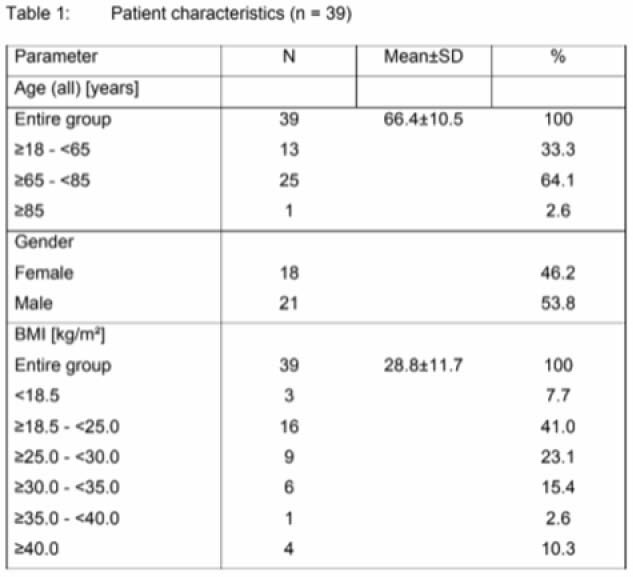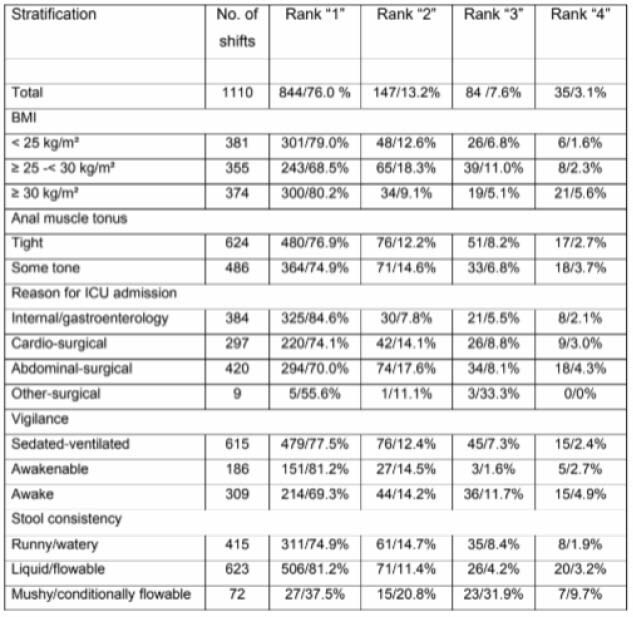# 880 Evaluation of a Novel Polyurethane-Based Fecal Management System: Implications for Burn Patient Care?

**DOI:** 10.1093/jbcr/iraf019.411

**Published:** 2025-04-01

**Authors:** Tobias Gutting, Keith Watts

**Affiliations:** Heidelberg University; Talexto Healthcare Partners

## Abstract

**Introduction:**

Fecal contamination poses a risk to burn patients, whose compromised skin barrier and immune function make them particularly susceptible to infections. Current silicone-based fecal management systems have limitations in sealing efficiency and anal tolerance. We hypothesized that a novel polyurethane-based fecal management system would demonstrate improved sealing efficiency and reduced incidence of anal lesions, potentially offering superior protection for burn patients against fecal-associated complications.

**Methods:**

This prospective, open-label observational study included 39 ICU patients requiring fecal management. While not specifically burn patients, the findings have important implications for burn care. The primary outcome was sealing efficiency, defined as the percentage of shifts without leakage. Secondary outcomes included the incidence of anal lesions and device-related adverse events. Data were collected over 1,110 documented shifts. Descriptive statistics were used to analyze outcomes.

**Results:**

The polyurethane-based system demonstrated a sealing efficiency of 89.3%, potentially reducing exposure to infectious agents in compromised patients. The incidence of anal lesions was remarkably low at 0.8%, an important finding for patients with extensive skin damage. No device-related adverse events were reported.

**Conclusions:**

The novel polyurethane-based fecal management system showed high sealing efficiency and a low incidence of anal lesions. For burn patients, these findings suggest potential advantages in reducing exposure to fecal contaminants and minimizing additional skin damage. The improved sealing and reduced lesion incidence could contribute to infection prevention in this vulnerable population. However, controlled trials specifically in burn patients are necessary to confirm these potential benefits and establish comparative efficacy in burn care settings.

**Applicability of Research to Practice:**

In burn care practice, this polyurethane-based system could reduce the risk of fecal contamination-related complications by potentially decreasing exposure to infectious agents and minimizing additional skin trauma. This system may contribute to improved wound healing and reduced infection rates in burn patients, though further studies are needed to confirm these benefits in this specific population.

**Funding for the Study:**

The manufacturer provided a grant to conduct the research in a university hospital setting.